# Exploring the global mosaic of medicinal plant databases: unveiling nature’s pharmacopoeia

**DOI:** 10.1007/s13659-026-00614-2

**Published:** 2026-06-01

**Authors:** Yifei Jiang, Xiaozhuan Jia, Zhenzhong Yang

**Affiliations:** 1 Pharmaceutical Informatics Institute, College of Pharmaceutical Sciences, Zhejiang University, Hangzhou, 310058 China; 2 Department of Biomedical Informatics, Yong Loo Lin School of Medicine, National University of Singapore, Singapore, 119228 Singapore; 3 Innovation Institute for Artificial Intelligence in Medicine, Zhejiang University, Hangzhou, 310018 China; 4 State Key Laboratory of Chinese Medicine Modernization, College of Pharmaceutical Sciences, Zhejiang University, Hangzhou, 310058 China

**Keywords:** Medicinal plants, Database, Geographical distribution, Natural products, Traditional medicine, Phytochemical information

## Abstract

Medicinal plants have long served as an important asset in the treatment of diseases. Recent developments in computer science have enabled the rise of specialized databases cataloging medicinal plant knowledge. However, a systematic comparison of available region-specific medicinal plant databases is lacking. This review summarizes globally available medicinal plant databases that focus on specific geographical regions, aiming to inspire and guide people from specialists to the general public toward fostering innovation and making informed decisions. Through a systematic search of literature and digital resources, 81 regional medicinal plant databases established or updated between 2013 and 2025 were identified. From this pool, 40 core platforms were subjected to detailed statistical characterization regarding their data categories and volume. Our analysis reveals a geographical concentration in Asia (48.1%), dominated by China and India, alongside a notable proliferation of universal databases with a global scope. These databases facilitate critical applications in drug discovery, quality control, biodiversity conservation, and policy-making. By identifying current research gaps and emphasizing the need for interdisciplinary standardization, this review serves as a strategic roadmap for bridging traditional wisdom with modern therapeutic innovation.

**Graphical abstract:**

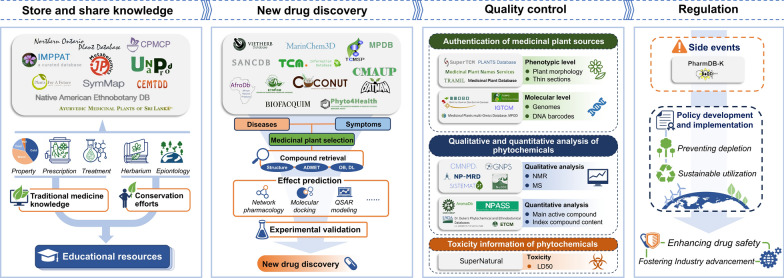

## Introduction

Plants represent a ubiquitous and predominant life form on Earth, exhibiting a wide-ranging distribution across diverse habitats encompassing the hydrosphere, lithosphere, and the atmospheric expanse. The botanical kingdom comprises an estimated 379,950 accepted species [1] serving multifarious purposes such as sustenance, pharmaceutical applications, timber production, dye synthesis, fiber extraction, and ornamental aesthetics [2]. In the realm of drug discovery, plants emerge as invaluable pioneers. Many of today’s ground-breaking pharmaceuticals, such as morphine, taxol, physostigmine, quinidine, emetine, and artemisinin, trace their roots to the botanical realm [3–5]. Throughout history, medicinal plants have been indispensable to human health [6], with the World Health Organization estimating that approximately 60% of the global population relies on them for primary healthcare [7].

Recent advances in computer science and network pharmacology have led to databases with advanced features, transforming contemporary medicinal plant research [8, 9]. Several review studies have evaluated medicinal plant databases, focusing on ethnopharmacology [10], computation [11, 12], bioinformatics [13–15], structural chemistry [16], bibliometrics [17], database-building approaches [18], and analytical overviews [2, 19, 20] of typical databases.

While medicinal plant databases are expanding, few articles have summarized medicinal plant databases oriented to specific geographical regions. The absence of interconnected information poses a significant barrier to understanding the cultural context of native plant use and the complex influence of environmental factors on plant metabolites. To address this, the present work provides a comprehensive bioinformatics perspective that bridges fragmented ethnobotanical records with the data-driven requirements of modern drug discovery. Guided by the FAIR (findability, accessibility, interoperability, and reusability) principles [21], we investigated a total of 81 freely accessible medicinal plant databases. While our selection primarily focused on platforms established or updated between 2013 and 2025, several foundational resources, i.e., BDTOI, HERBA, SWMD, and the Native American Ethnobotany Database, were also integrated to ensure the comprehensiveness of the survey.

**Fig. 1:**
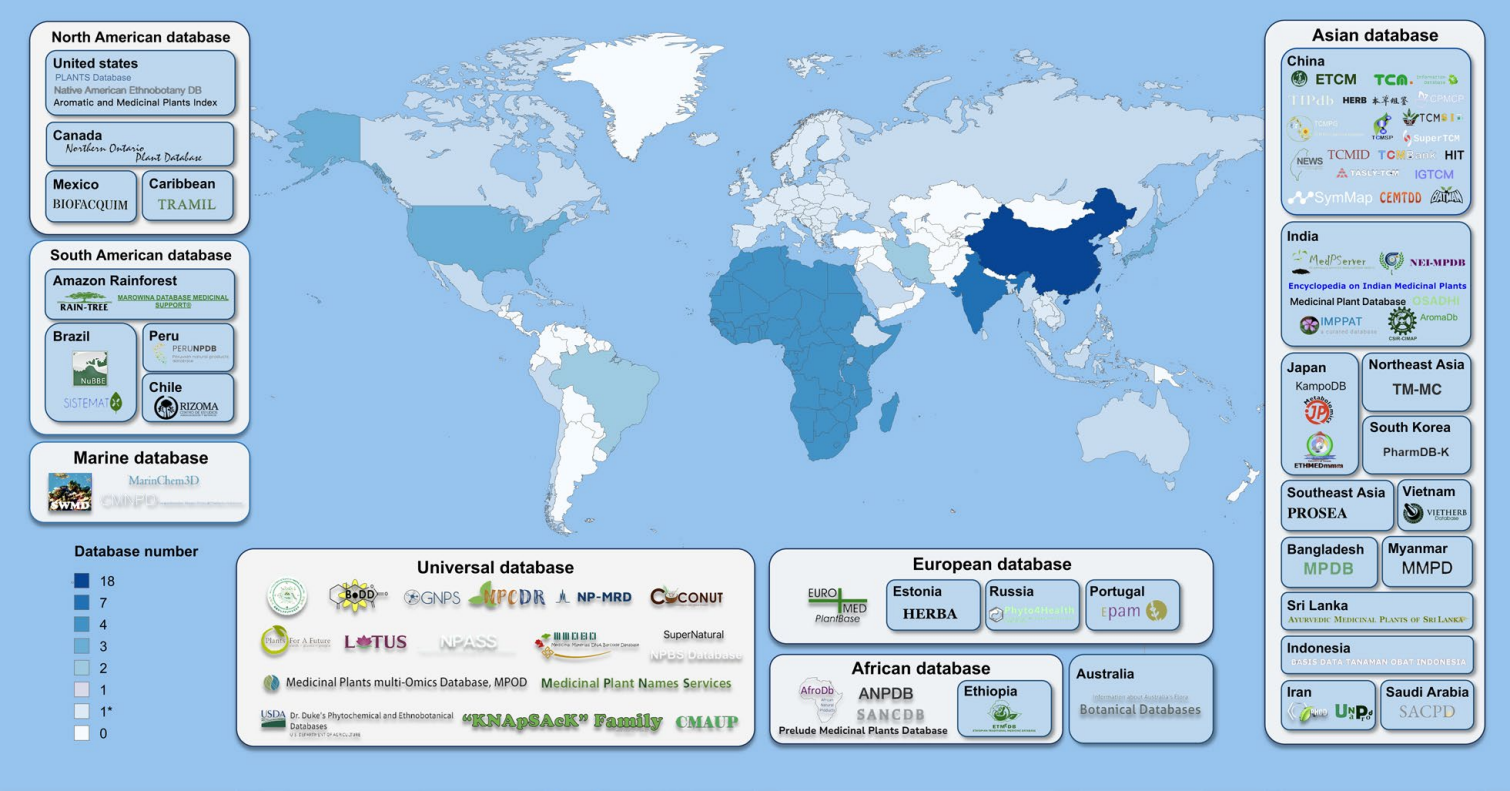
Geospatial analysis of medicinal plant databases developed or updated between 2013 and 2025. Various colors on the map correspond to the number of databases in distinct geographic regions. The asterisk (*) denotes databases that cater to multiple countries and regions

As shown in Fig. 1, it appears that most databases are produced for recording Asian (48.1%) plants, especially China (22.2%) and India (8.6%). Universal (21.0%) databases like COCONUT [22, 23], SuperNatural [24], and NPASS [25] have gained significant popularity among researchers due to their extensive collection of data on medicinal plants from around the world. Latin America stands out for its rich and unique biodiversity, and some open databases (9.9%) have gathered and characterized its medicinal plants, with particular attention to the Amazon rainforest and the Caribbean. Also, several countries are collaborating to build a unified database LANaPD [26], which aims to represent the whole biodiversity of Latin America. There are five typical African (6.2%) medicinal plant databases, including ANPDB [27], SANCDB [28] and so on. ANPDB is now under construction to compensate for the lack of records of West African natural products. Both North America (4.9%) and Europe (4.9%) have four databases representing their medicinal plant diversity. Some databases (3.7%) are built for marine natural products, including compounds derived from marine plants and other organisms [23]. For now, there is a minimal quantity of databases from the Australian continent (1.2%), only CANBR and ANBG botanical databases integrated Australian taxonomic botany and plant species information.

In fact, the reasons for the wide global variation in medicinal plant prevalence are traceable, and they could be attributed to cultural background, healthcare systems, availability of medicinal plants, and economic conditions. Traditional East Asian medicine, originating around 3000 years ago in China and subsequently introduced to Korea and Japan in the sixth century, has been deeply ingrained in these societies, forming a significant part of their healthcare practices [29]. In China, for instance, traditional Chinese medicine (TCM) is integral to medical care, with 95% of general hospitals housing specialized TCM departments, utilizing it extensively for treatment and prevention [18]. Similarly, in Japan, over 80% of medical practitioners prescribe Kampo medicines, showcasing a widespread acceptance of traditional healing methods [30]. In India, AYUSH is the acronym for Ayurveda, Yoga and Naturopathy, Unani, Siddha, Sowa-Rigpa, and Homeopathy, representing a structured healthcare segment that extends its services across both public and private sectors [18]. Europe, as the world’s second-largest market for complementary and alternative medicine (CAM) after Asia [31], demonstrates varying utilization rates across countries, ranging from 10% in Hungary to nearly 40% in Germany [32]. Furthermore, the availability of medicinal plants varies depending on the climate, soil, and other environmental factors [33]. Studies [34–36] have illuminated a social connection between the utilization of medicinal plants and prevailing economic circumstances.

Medicinal plants represent a valuable resource for human health, serving as a wellspring of natural compounds that have been utilized in traditional medicine for centuries. As we navigate the evolving landscape of healthcare and pharmacology, the synergy between traditional knowledge, advanced technologies, and artificial intelligence (AI) is becoming increasingly significant. The availability of medicinal plant databases, coupled with the power of AI, is reshaping the way we explore, understand, and apply these natural remedies. In the upcoming sections, we will delve into a comprehensive exploration of these identified resources and examine their specific attributes and multifaceted applications. These databases offer a wealth of knowledge, inspiring and guiding professionals and researchers in diverse fields, including healthcare, ethnobotany, AI, and environmental science, to drive innovation and informed decision-making.

## Comparative studies of available medicinal plant resources

With an estimated total of 379,950 plant species identified globally [1], the potential number of medicinal plants represents a proportion ranging from 13 to 35% [37]. These medicinal floras exhibit a wide geographic distribution across diverse biomes and are considered a rich source of specialized metabolic compounds known as phytochemicals. Numerous studies [38–42] have demonstrated that phytochemicals have the ability to influence a broad range of bodily functions, thereby conferring therapeutic efficacy against an array of human pathologies, and this is precisely the basis on which these medicinal plants demonstrate their therapeutic effects in treating diseases.

**Fig. 2:**
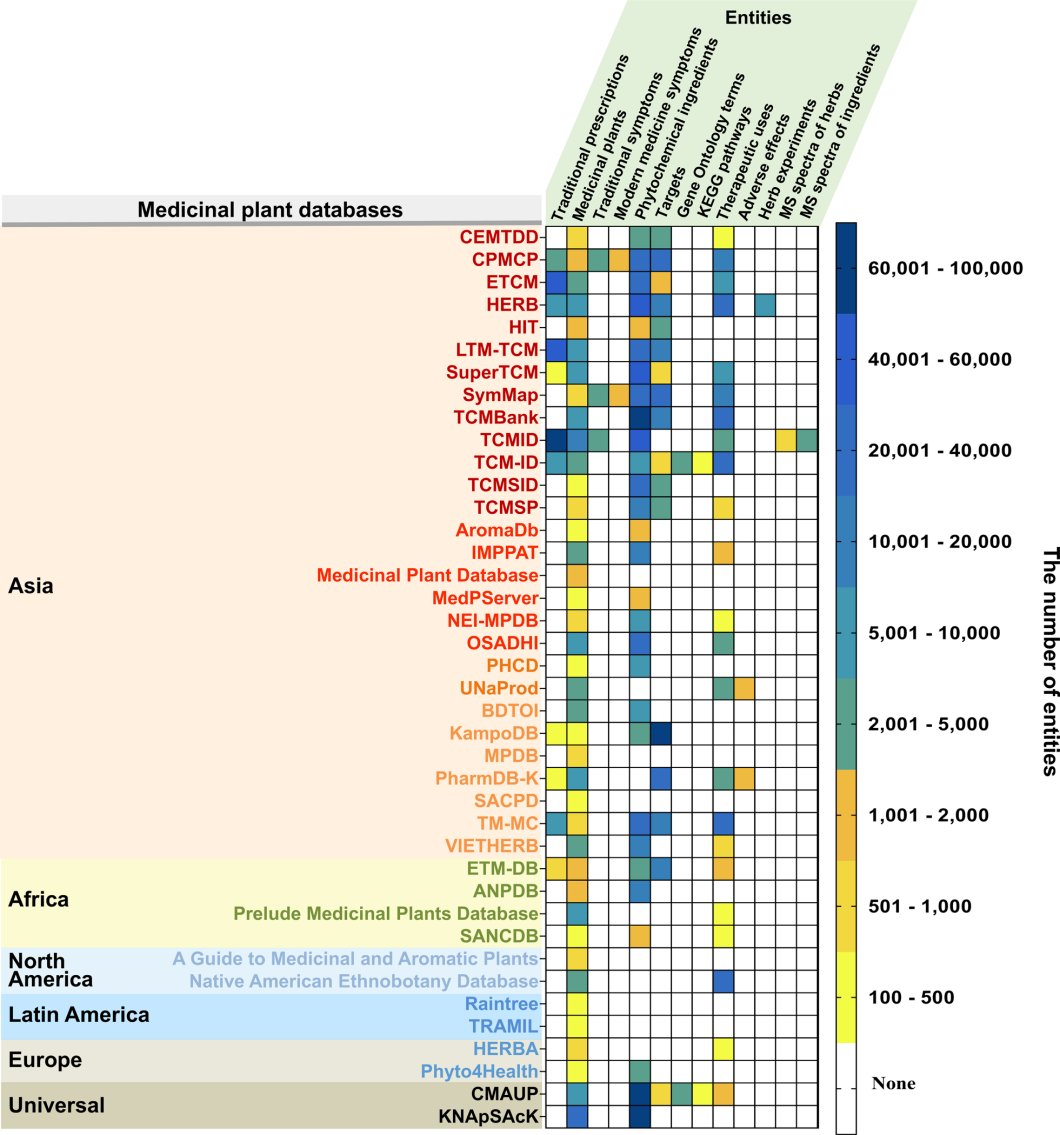
Heat map illustrating 40 medicinal plant databases across continents and entity composition. In the Asia section, the names of the databases in four colors, arranged from top to bottom, represent China, India, Iran, and other Asian regions respectively

To further explore the global distribution of medicinal plants, we generated a heat map as depicted in Fig. 2. This visualization helps to identify how data from different regions contribute to our understanding of medicinal plant diversity and their therapeutic potential. We focused our study on 40 out of the 81 available global databases, selecting them based on criteria that ensure broad geographic coverage, adherence to proportional representation among similar databases, and the availability of specific entity statistics. The heat map elucidates the relative quantities of collated data entities such as plant species, prescriptions, phytochemical ingredients, genes, KEGG pathways, therapeutic applications, adverse effects, and so on. Each database offers a unique blend of these entities, presenting a comprehensive snapshot of medicinal plant knowledge that spans different regions and countries.

To gain a more profound comprehension of the construction, content, and utilization of medicinal plant databases, our focus centered on an investigative analysis of distinctly characterized databases. This scrutiny allowed us to delve into their respective attributes and synthesize pivotal information, collating it into Table 1 for comparative observation.

**Table 1:** Summary of 19 typical medicinal plant databases with their properties

No.	Database	Plants Location	Continent	URL and Description	Last Update	First Launch	Language	Statistics
1	Prelude Medicinal Plants Database [43]	Africa	Africa	http://www.africamuseum.be/collections/external/prelude A comprehensive database documenting plants utilized in traditional veterinary and human medicines across Africa	2019	1996	English	Plants: 8152 Human symptoms: 333 Veterinary symptoms: 156 African countries: 54
2	BATMAN-TCM [44, 45]	China	Asia	http://bionet.ncpsb.org.cn/batman-tcm/#/home An online bioinformatics analysis tool specifically crafted for investigating the molecular mechanisms underlying TCM	2023	2016	English	Formulas: 54,832 Herbs: 8404 Compounds: 39,171 Target proteins: 9927 Pathways: 217 Diseases: 5128
3	ETCM [46, 47]	China	Asia	http://www.tcmip.cn/ETCM2/front/#/ A structured and high-quality TCM database with multi-source data integration	2022	2019	English, Chinese	TCM formulas: 48,442 Chinese patent drugs: 9872 Chinese medicinal materials: 2079 Ingredients: 38,298 Targets: 1040 Diseases: 8045
4	HIT [48, 49]	China	Asia	http://hit2.badd-cao.net A literature-based search and curation platform for herbal ingredients and associated biological targets	2021	2010	English	Source herbs: 1250 Herbal ingredients: 1237 Biological targets: 2208
5	IMPPAT [50, 51]	India	Asia	https://cb.imsc.res.in/imppat/ A meticulously curated database encompassing information on Indian medicinal plants, phytochemistry, and therapeutic applications	2022	2018	English	Medicinal plants: 4010 Phytochemicals: 17,967 Therapeutic uses: 1095
6	OSADHI [52]	India	Asia	https://neist.res.in/osadhi/ An Indian database of medicinal plants with their traditional knowledge, geographical classification, and phytochemicals information	2023	2022	English	Medicinal plants: 6959 Phytochemicals: 27,440 Therapeutic uses: 2477 Indian states/union territories: 38
7	UNaProd [53]	Iran	Asia	https://unaprod.com/ A comprehensive natural product database encompassing Iranian traditional medicine	2020	2020	English, Farsi, Arabic, Chinese, Kannada, Hindi, Urdu	Iranian drugs: 3413 Medicinal uses: 2610 Adverse effects: 1114
8	KampoDB [30]	Japan	Asia	https://wakanmoview.inm.u-toyama.ac.jp/kampo/ An innovative database designed for the analysis of traditional Japanese medicines	2023	2018	English	Kampo formulas: 298 Crude drugs: 180 Natural compounds: 3002 Proteins/genes: 62,906
9	VIETHERB [54]	Vietnam	Asia	https://vietherb.com.vn/ A database of traditional Vietnamese medicine	2016	2016	Vietnamese, English	Herbal species: 2828 Herbal metabolites: 10,239 Therapeutic effects: 639
10	Phyto4Health [55]	Russia	Europe	http://www.way2drug.com/p4h/ A secondary metabolites database of Russian official medicinal plants	2021	2021	English	Medicinal plants: 268 Phytocomponents: 3128 Records of biological activity in compounds in vitro: 13,688 Records of biological activity in compounds in silico: 1,332,884
11	NuBBE [56, 57]	Brazil	Latin America	https://nubbe.iq.unesp.br/portal/nubbedb.html An updated platform aimed at revealing chemical and biological insights derived from the rich biodiversity of Brazil	2017	2013	English, Brazilian	Phytoconstituents: 1688 Brazilian states: 24
12	Raintree [58]	Amazon Rainforest	Latin America	https://rain-tree.com/plants.htm A database of Amazonian plant species integrating taxonomic, phytochemical, traditional use, and clinical trial information	2019	1996	English	Medicinal plants: 232 Diseases: 302
13	CMNPD [59]	Marine	Marine	https://www.cmnpd.org/ An open access, literature-based database cataloging information on marine natural products	2020	2020	English	Compounds: 31,561 Organisms: 3354 Targets: 2652
14	Native American Ethnobotany Database [60]	North America	North America	http://naeb.brit.org/ A database documenting plants employed by Native people of North America for their medicinal, culinary, dyeing, and fiber-related properties	NA	1977	English	Plant species: 4260 Native American tribes: 291 Therapeutic uses: 24,945
15	COCONUT [22, 23]	Universal	Universal	https://coconut.naturalproducts.net/ An open-access bioinformatics database for natural product structures and analyses	2025	2020	English	Natural products: 695,117
16	GNDC [61]	Universal	Universal	https://cbcb.cdutcm.edu.cn/gndc/ A database dedicated to capturing gene-encoded natural diverse components sourced from herbal medicines	2025	2025	English	Medicinal plants: 1037 Secondary metabolites: 2,320,000 + Peptides: 229,770,000 + Small RNAs: 2,380,000 + Carbohydrate: 260,000 +
17	GNPS [62]	Universal	Universal	http://gnps.ucsd.edu An online platform for the analysis and sharing of mass spectrometry data from natural products	2025	2014	English	NA
18	KNApSAcK [37]	Universal	Universal	http://kanaya.naist.jp/KNApSAcK_Family/ An integrated database cataloging metabolites derived from plants, organized based on their geographic zones	2024	2008	English, Japanese	Medicinal plants: 24,749 Countries: 229 Plant metabolites: 63,715
19	MPNS [63]	Universal	Universal	https://mpns.science.kew.org/mpns-portal/version A global resource that provides information on the scientific and common names of medicinal plants	2024	2014	English	Plants: 39,112 Plant families: 393 Plant scientific names: 59,470 Plant non-scientific names: 271,207 Medicinal data sources: 396

The list is ordered by alphabetical order of the continent, plants location or database names

### Prelude medicinal plants database

Prelude Medicinal Plants Database is dedicated to the exploration of plant usage in various traditional veterinary and human medicines across Africa. Users can navigate the database by plant, country, symptom, and reference, with information sourced from scientific articles, books, papers presented at congresses, or contributed directly to the Prelude Sub-Network on “Health, animal productions, environment”. For researchers, doctors, veterinarians, and agricultural instructors, this database can help them quickly find information on the use of African medicinal plants for therapeutic purposes through a reliable literature guidance.

### BATMAN-TCM

BATMAN-TCM is designed to help researchers and practitioners better understand the complex and holistic nature of TCM by providing a systematic and integrative approach to analyze the relationships between TCM formulas, herbal ingredients, and disease targets at the molecular level. Its version 2.0 significantly expands the coverage of known and predicted interactions between TCM ingredients and target proteins. The database integrates a repository of over 17,000 manually curated interactions from literature and external databases, complemented by approximately 2.3 million high-confidence predictions generated via a similarity-based method. In addition to a novel retrieval function that screens herbs based on disease signature genes from omics studies, BATMAN-TCM enables users to input TCM formulas or TCM herbs to generate predicted target profiles, network maps, and pathway annotations based on the available experimental data and computational models. The tool also allows for the discovery of novel combinations of TCM herbs that could have synergistic effects.

### ETCM

ETCM is an important bridge between TCM and modern medical research. It provides multi-dimensional intelligent correlation analysis and graphical presentation of “TCM formulas-Chinese medicinal materials-ingredients-genes-functions/pathways-diseases”, comprehensively analyzing the interaction between the complex system of TCM and the molecular network of the body. ETCM has structured and standardized the information of TCM ancient formulas and Chinese patent drugs, which has more advantages than other similar TCM databases. It has also established external links with international authoritative chemical and biological databases such as PubChem, UniProt, and GeneCards.

### HIT

HIT is a literature-based search and curation platform containing information on over 1250 herbal medicines, 1237 extracted ingredients, and 2208 associated biological targets. The target database catalogs proteins and enzymes that are directly or indirectly activated, inhibited, or bound by herbal compounds. The platform also provides My-Target, a curation system to review compound-target relationships and build customized profiling of the latest research. Overviews and detailed records foster exploration of polypharmacology within traditional remedies and discovery of new bioactivities.

### IMPPAT

IMPPAT is an open-access database cataloging over 4000 Indian medicinal plant species, nearly 18,000 associated phytochemicals, and 1100 therapeutic applications. Curators have manually compiled data from more than 100 traditional medicine texts, 7000 + scholarly research articles, and other resources. This up-to-date literature-based platform allows exploration of individual plants, compounds, or medicinal uses. Users can access details on phytochemical properties, structures, and absorption, distribution, metabolism, excretion, and toxicity (ADMET) profiles to facilitate natural product drug discovery and elucidate mechanisms of action. By integrating information on India’s rich botanical heritage and linking compositions to effects, IMPPAT aims to accelerate investigations into phytochemistry and target validation, supporting both computational and empirical phytomedicine research.

### OSADHI

OSADHI is an open-access database cataloging over 6900 medicinal plant species from across India. This database offers detailed records on plant taxonomy, ethnobotanical uses, physiological sources, isolated phytochemicals, and reported therapeutic applications. Detailed analysis for the 22,314 phytochemicals has been provided in this database which includes physicochemical properties, drug-likeness (DL) based on multiple scoring schemes, predicted ADMET properties, and predicted antiviral potency. It provides an integrated platform for using chemoinformatic methods to hasten the development of drugs based on natural products.

### UNaProd

UNaProd is an open-access database cataloging over 3400 medicinal substances within Iranian traditional medicine (ITM). Individual monographs provide comprehensive documentation across 16 attributes, including identity, temperament classification, medicinal actions, adverse effects, preparation methods, origins and more. Key information on each natural remedy such as *Mizaj* categorized similarly to TCM as cold-dry, hot-wet, etc. aims to preserve the holistic doctrines of ITM. The database also links to related resources on molecular signatures and an ITM ontology. By integrating diverse data on the natural products, practices and conceptual foundations of ITM, UNaProd supports both basic and translational investigations into Persian ethnomedicine. It serves to elucidate mechanisms of complex herbal formulations while safeguarding integral aspects of this important medical tradition.

### KampoDB

KampoDB is an open-access resource for exploring Kampo medicine, a traditional Japanese system adapted from Chinese herbalism. With centuries of clinical use, Kampo formulations address diverse conditions. The database provides scientific information on Kampo prescriptions, raw herbs, constituent phytochemicals and associated protein targets. Users can search individual medicines to uncover properties, molecular effects and potential new therapeutic applications through protein interaction networks. By integrating compound data with large-scale omics profiles, KampoDB also predicts undiscovered targets of natural product constituents. This elucidates mechanisms of complex formulas while supporting research into multi-target effects. Overall, the platform advances Kampo therapy and phytomedicine by linking formulations to biological pathways and facilitating both target validation and systems-level investigations into the medical tradition.

### VIETHERB

VIETHERB is an open-access database documenting over 2800 plant species, 10,000 metabolites, and traditional uses from Vietnamese herbal medicine. Users can explore profiles detailing individual herbs with metadata on geographical distribution, isolated compounds and therapeutic applications. Comprehensive records integrate ethnobotanical knowledge with phytochemical and pharmacological data. This curated platform facilitates a diversity of research avenues including modernization of Vietnamese medical practices, computer-aided drug discovery, computational target prediction and conservation science. By preserving ethnomedicinal wisdom while enabling omics technologies, VIETHERB aims to support validation and development of herbal therapies as well as conservation of medicinal flora.

### Phyto4Health

Phyto4Health is a database that compiles information on various biologically active compounds from 268 medicinal plants listed in the Russian Pharmacopoeia. The database contains information on 3128 phytocomponents, including their source plants, physical–chemical characteristics, and biological effects. The records of biological activity in compounds were obtained from the data of interactions with human molecular targets and prediction of activity spectra for substances, respectively. The overlap of phytocomponents within this database is comparatively limited in contrast to five other similar databases (NANPDB [64], SistematX [65], Ayurveda [66], BIOFACQUIM [67], and TPPT [68]), thereby significantly enhancing the uniqueness of the contents.

### NuBBE

NuBBE is a useful tool for studies on naturally occurring Brazilian bioactive compounds. It contains a variety of natural products isolated from Brazilian biodiversity and provides information on chemical (metabolic classes, chemical structures, physicochemical properties, etc.), biological (species, geographic locations, biological activities), pharmacological properties, and spectroscopic data. Its user-friendly interface and extensive search capabilities make it easy to explore the database and access the information needed for a wide range of research projects. Especially, NuBBE can be used to uncover the relation between Brazilian species with biological activities and geographical disposition.

### Raintree

Raintree has been working to provide accurate and factual information about important plants in the Amazon Rainforest. It can be browsed by plant common names, botanical names, ethnic uses, diseases, and properties. Information on herbal properties, utilized parts, dosages, plant chemicals, tribal uses, global ethnomedical uses, biological activities, clinical research, and current practical applications is provided. This aims to facilitate easy access to available plant information for both professional readers and newcomers to the field of medicinal plants. There are also links to various articles related to plants in the database, offering further insights and knowledge.

### CMNPD

CMNPD is a meticulously curated repository focused on marine natural products. Recognizing the vastness of the oceans covering 71% of Earth’s surface and the scarcity of open, noncommercial databases for marine natural products, CMNPD offers an extensive array of data on a wide range of chemical compounds, featuring various physicochemical properties and pharmacokinetic profiles. Alongside, it provides standardized biological activity data, systematic taxonomy, geographic distribution of source organisms, and spectral information including infrared spectroscopy (IR), mass spectrometry (MS), nuclear magnetic resonance (NMR), and ultraviolet–visible spectroscopy (UV/VIS). Detailed literature citations further enrich the resource. With the potential for future expansion, CMNPD aims to evolve into an even more comprehensive repository for marine natural products.

### Native American ethnobotany database

The Native American Ethnobotany Database is a digital repository of information about the traditional uses of plants by indigenous peoples of North America. The database consists of over 44,000 plant uses for purposes such as medicine, food, fiber, and ceremonial activities, with about half of these uses being medicinal. The information is drawn from over 1000 sources, including ethnographic field notes, historical documents, and published literature. The database is searchable by plant name, common name, tribe, and use, including detailed information on the cultural context of each use, as well as the scientific name and other taxonomic information for each plant. The Native American Ethnobotany Database is an important resource for researchers, educators, and indigenous communities interested in preserving traditional knowledge of plants and their uses. It serves as a valuable tool for understanding the rich cultural heritage of native people in North America and their relationship with the natural world.

### COCONUT

COCONUT is an open-source initiative designed for the storage, search, and analysis of natural products. In the 2.0 version released in 2025, the database expanded its data sources and significantly increased the number of unique natural product structures, while upgrading the data model to a stereochemistry-aware one. The latest version not only enhanced the database’s technical architecture and data standardization pipeline but also introduced community-driven curation features, enabling users to continuously contribute to data refinement and improvement, thereby enhancing the platform’s flexibility and sustainability. To date, the database comprises over 695,133 unique natural product structures, more standardized than before, from over 60 open-access natural product resources, among which 539,350 molecules have preserved stereochemical information. Associated natural product structure, predicted bioactivities, known stereochemical forms, literature, and diverse pre-computed molecular properties are provided annotating by a 5-star-based level system (5 stars is the highest quality). More, biological occurrences of 276,518 natural products in COCONUT can be found in the LOTUS [69] database. Developed by the same team as COCONUT, LOTUS has successfully harmonized, curated, validated, and openly disseminated over 750,000 referenced structure-organism pairs.

### GNDC

Gene-encoded Natural Diverse Components Repository (GNDC) is the first database dedicated to capturing gene-encoded natural diverse components sourced from herbal medicines. GNDC comprises four sub-databases, i.e., HerbalMDB (2.32 M metabolites), HerbalPDB (229.77 M peptides), HerbalRDB (2.38 M small RNAs), and HerbalCDB (0.26 M carbohydrates), covering 234 million components from 1037 species recorded in eight authoritative pharmacopoeias. Powered by an integrated transformer-GNN ADMET 2.0 pipeline, GNDC automatically embeds gene-encoded components into a unified chemical-biological latent space, predicts 25 druggability end points with AUC ≥ 0.89, and delivers ranked, clinic-ready candidates through a one-click web interface, thereby offering a "chemical-space"-grade operating system for natural-product drug discovery. GNDC addresses the limitations of existing natural product databases in data types and functional analysis, accelerates big-data-driven drug discovery, and represents a key future tool for exploring natural products with AI technology.

### GNPS

GNPS is a web-based platform that allows scientists to analyze and interpret MS data of complex mixtures of natural products. This platform was developed to aid in the discovery and identification of new bioactive chemical entities from natural sources. It organizes the MS data into a network of nodes and edges where each node represents a molecule and each edge represents the structural similarity between two nodes. This helps in visualizing the relationships between different molecules in a sample. It also includes a vast library of spectra from known molecules which can be used by scientists to compare their data with this library to identify known compounds in their samples. One of the unique features of GNPS is its social networking aspect, which allows researchers to collaborate and share data with each other.

### KNApSAcK

KNApSAcK is a comprehensive database that describes the relationships between plants and their metabolites. Updated to 24 December 2024, KNApSAcK WorldMap is a database of world-wide utilization of 24,749 medicinal plant species sorted by 229 individual countries that have been collected and registered from published scientific reports. KNApSAcK provides comprehensive search and exploration tools for metabolomics research. Its MS-focused search engine allows users to query metabolites by accurate mass, molecular formula, name or mass spectra across ionization modes. Included within KNApSAcK is the Biological Activity DB, also enables investigations of medicinal plants. Researchers can uncover each species’ reported biochemical activities, associated disease states and human health impacts.

### MPNS

Kew’s Medicinal Plant Names Services (MPNS) is a database that provides standardized and curated information on the names of medicinal plants used in traditional and modern medicine around the world. The MPNS database contains information on all pharmaceutical, herbal drug, common and scientific plant names as cited for plants or herbal drugs in any of the medicinal plants and health regulatory literature. It also includes information on the plant’s used parts and drug forms. One of the unique features of MPNS is its focus on providing accurate and up-to-date information on the names of medicinal plants, including taxonomic changes and updates.

## Applications from traditional remedies to cutting-edge discoveries

Databases focused on national or regional contexts often mirror the distinct requirements of particular communities [10]. Although these databases are contextualized to offer invaluable local insights, their underlying principles of development and application remain universal. These databases themselves serve as de facto guidance for global collaboration, uniting researchers, clinicians, academics, and various stakeholders. They facilitate the exchange of knowledge, techniques, and experiences, thereby fostering a cohesive environment for utilizing and augmenting medicinal plant databases.

**Fig. 3:**
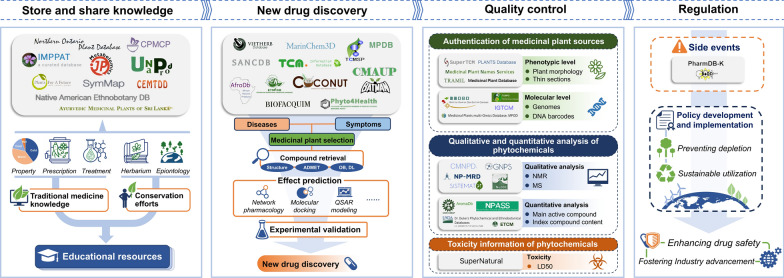
Integrative applications of medicinal plant databases. Medicinal plant databases facilitate the compilation and sharing of traditional medicine knowledge, enable the discovery of new drugs from natural products, ensure the quality control of herbal medicines, and assist in the regulation of herbal medicine practices

The versatile nature of medicinal plant databases extends far beyond mere storage of information. Their significance spans various domains (shown in Fig. 3), serving as reservoirs of traditional knowledge, catalysts for pharmaceutical innovation, guarantors of product quality, and regulators of herbal medicinal usage. Embracing these databases as shared resources paves the way for a collaborative ecosystem benefiting diverse stakeholders worldwide.

### Store and share knowledge

A medicinal plant database serves as an information bank that effectively stores and shares knowledge with the community. It helps support scientific research by extensively cataloging information on medicinal plants.

#### Traditional medicine knowledge

Traditional medicine practices, such as Ayurveda, TCM, Unani medicine, and ITM, heavily rely on the use of medicinal plants. Databases can serve as valuable resources for practitioners in these fields, helping them identify and learn more about the plants used in their practice.

In UNaProd [53], each drug monograph is dedicated to *Mizaj* (temperament, a concept in ITM), and database statistics show that for monotherapy in *Makhzan al-Advieh*, the most common *Mizaj* type was hot-dry (55.7%), followed by cold-dry (16.5%), hot-wet (8.1%), and cold-wet (5.4%).

In China, many people use TCM prescriptions which consist of several TCM medicinal materials to treat complex diseases. For example, *Fangjihuangqi* decoction, which contains the roots of *Stephania tetrandra* S. Moore, the roots of *Astragalus membranaceus* (Fisch.) Bge. var. *mongholicus* (Bge.) Hsiao, the rhizomes of *Atractylodes macrocephala* Koidz., and the roots and rhizomes of *Glycyrrhiza uralensis* Fisch., is used for the treatment of chronic heart failure [70, 71]. CPMCP [72] is the latest curated database to provide such information about 2125 TCM prescriptions and summarizes a set of common drug combination principles by analyzing the combination of medicinal materials in existing prescriptions. Another characteristic of TCM is symptom differentiation and treatment. SymMap [73] categorizes over 2518 TCM symptoms according to their meridians in the body and properties.

When developing traditional knowledge databases like the Native American Ethnobotany Database, integrating indigenous knowledge requires a harmonious and respectful approach under the CARE principles (collective benefit, authority to control, responsibility, and ethics) [74]. This includes emphasizing the protection of local rights and resources, which may be affected or disrupted by external interests. The development of such databases should involve indigenous communities collaboratively and in an orderly manner, ensuring that their rights and resources are not infringed upon, but preserved and enhanced [75]. In the case of the Aurukun ethnobiology database project [76], Aboriginal communities co-developed the database with ethnoscientists through a participatory research process that adhered to indigenous methodologies and cultural protocols. Despite concerns expressed by some ethnoscientists [77, 78] who argue against similar future projects, this collaboration stands as a good example of respecting and empowering community control over data and knowledge management. This debate underscores the need for research approaches that both respect indigenous rights and advance community-centered goals.

#### Conservation efforts

Medicinal plant databases are valuable tools for conservationists and environmentalists to identify endangered species and prioritize their conservation efforts. The Ayurvedic Medicinal Plants of Sri Lanka website (http://www.ayurvedicmedicinalplantssrilanka.org/) supports biodiversity conservation by providing comprehensive data on the taxonomic rank, geographical localization, and therapeutic potential of plants. Such databases, as emphasized by the Global Strategy for Plant Conservation under the Convention on Biological Diversity [79], are crucial for achieving global conservation targets. Additionally, the integration of geographic information system (GIS) technology enhances these efforts by offering detailed mappings of plant species distributions, critical for effective conservation planning and implementation [80].

#### Educational resources

Students and professionals in the fields of pharmacy, medicine, and botany can benefit from medicinal plant databases as educational resources. These databases not only serve as reference materials for traditional uses and resource conservation, but also provide integrated information from both traditional and modern perspectives. For example, databases like SymMap [73] offer insights into TCM by correlating TCM symptoms with modern symptoms. This enables the delivery of comprehensive educational content, combining traditional knowledge systems with modern scientific research to facilitate a deeper understanding of plant-based medicine.

Medicinal plant databases can help raise public awareness about the benefits and potential risks associated with the use of medicinal plants. By providing accurate and accessible information, these databases can empower individuals to make informed decisions about their health and well-being.

### New drug discovery

Medicinal plant databases serve as not only integral components of traditional medical systems, but also as viable, cost-effective strategies and tools in the realms of chemo- and bioinformatics for novel drug discovery.

#### Screening for lead compounds

The search for new drugs from phytomedicine usually begins with the identification of diseases and corresponding symptoms, guiding the selection of relevant medicinal plants. In this initial stage, universal databases such as COCONUT [22, 23] and NPASS [25] are invaluable, providing extensive data on physicochemical properties, DL, or ADMET characteristics of natural compounds. For in-depth research on medicinal plants from specific regions, databases tailored to such locales are crucial. For instance, SANCDB [28, 81] catalogs South African natural compounds, while ETM-DB [82], focuses on Ethiopian herbs. These databases provide detailed insights into the biochemical profiles and therapeutic potentials of local flora, enriching the preliminary screening process, and enhancing the discovery of bioactive molecules.

Network pharmacology validation, through molecular biology experiments, predicts targets and provides guidance for exploring effective targets for the active compounds [83]. Specifically, TCM network pharmacology establishes a “compound-target-disease” relationship [84–86], which can be used to explore the complex associations between medicinal plants and diseases. Through network pharmacology, Chen et al. [87] used TCMSP [88] and TCM-ID [89] to identify potential active compounds and their targets in fruits of *Euodia rutaecarpa*, utilizing both 2-dimensional and 3-dimensional quantitative structure activity relationship (QSAR) models to predict and evaluate their interactions with biological targets. Additionally, molecular docking techniques are used to confirm the binding efficiency and modes of these compounds at the atomic level, providing insights into their potential efficacy.

In recent years, as chemical libraries have expanded to exceed a billion compounds, AI-enabled virtual screening approaches leveraging deep learning for molecular docking have emerged as economical and highly reliable techniques for evaluating very large datasets, which has been successfully applied using ZINC20 database [90].

#### Structure optimization of lead compounds

Morstein et al. [91] applied COCONUT for chemoinformatic characterization of the “Natural Product Lipidome”, analyzing lipid chain length, hydrogen bond acceptor count, connecting functional groups, and varying degrees of saturation, which revealed the conjugation of small molecules with medium-chain lipids could improve cell permeability and bioactivity of small molecules.

### Quality control of herbal medicines

#### Authentication of medicinal plant sources

The traditional authentication of plant sources can be started from three aspects: observing plant morphology, verifying literature descriptions, and examining specimens. The Medicinal Plant Database developed by the Indian government (https://bsi.gov.in/page/en/medicinal-plant-database) contains herbarium images and other details of Indian medicinal plants that can aid identification. SuperTCM [92] is another useful resource, featuring a comprehensive collection of high-quality photographs of authentic Chinese herbs to support research. The TRAMIL library (https://www.tramil.net/en/content/discover-the-tramilibrary) not only offers photographs and specimens of medicinal plants from the Caribbean region, but also provides thin sections stained with carmine-green and photographed using a light microscope. Images are crucial for proper species identification, which is the foundation for determining a plant’s medical applications and safety.

While phenotypic characteristics provide important clues, the precise identification and taxonomic classification of plant species often require complementary analysis at the molecular level [93]. MPOD [94] is an open-access multi-omics database integrating diverse genomes datasets compiled from public repositories and proprietary research. MMDBD [95, 96] provides DNA barcodes, such as rbcL, matK, nuclear ribosomal ITS, ribosome DNA, and other plastid DNA regions for medicinal plants identification and authentication.

#### Qualitative and quantitative analysis of phytochemicals

Medicinal plants contain a variety of active phytochemicals, which are related to complex secondary metabolic pathways. NMR and MS have unique advantages in the identification of phytochemicals [97, 98]. SistematX [65, 99, 100] generates and visualizes ^1^H and ^13^C NMR data, and implements the prediction of activity spectra for substances tool directly. GNPS [62] shares raw, processed, or annotated fragmentation mass spectrometry data (MS/MS) offering retrieval and reanalysis for natural products.

Quantitative analysis of active compounds is essential for understanding their efficacy, forming the foundation for the quality control. For the evaluation of TCM quality, ETCM [46, 47] provides standardized quantitative criteria for index compounds of TCM. Furthermore, techniques such as chromatography-mass spectrometry offer even more detailed insights by combining separation capabilities with mass spectrometric detection, thus providing a comprehensive analysis of the complex mixtures often found in medicinal plants [101]. AromaDb [102] archives extensive gas chromatography-mass spectrometry (GC–MS) analysis data for medicinal and aromatic plants. Additionally, Dr. Duke’s Phytochemical and Ethnobotanical Databases (https://phytochem.nal.usda.gov/) provide another essential layer of insight by enabling users to pinpoint which plants exhibit the highest concentrations of specific phytochemicals.

#### Toxicity information of phytochemicals

Understanding toxicity is equally crucial in quality control processes. SuperNatural 3.0 [24] significantly aids in this regard by providing potential toxicity alerts for the use of specific phytochemicals.

### Regulation of herbal medicines

Safety assessment is a critical part of regulation. For example, databases such as PharmDB-K [103] report side effects of various botanicals to inform this process.

Regulatory oversight extends beyond drug quality control to include crucial areas such as drug policy development and implementation. Legislation for herbal medicinal products, dietary supplements, and nutraceuticals varies between regions like China, Japan, the US, Australia, New Zealand, Canada, and the EU [104]. Given this variability, medicinal plant databases can serve as a tool to help harmonize regulatory frameworks for botanicals internationally. This enables the formulation of rational policies that ensure sustainable use of plant resources by preventing depletion, as well as facilitating the development of new evidence-based herbal medicines. An optimal regulatory approach should encompass both public safety and the conservation of medicinal botanicals.

## Conclusions and perspectives

Medicinal plants remain indispensable for global health, and the rapid evolution of computer science has positioned digital databases as vital repositories for this botanical heritage. Following a systematic screening of the current database landscape, this review provides a comprehensive evaluation of regional medicinal plant resources established or updated over the last 12 years, with a curated focus on 19 representative platforms selected for their structural depth and functional impact. By offering a bioinformatics perspective on global botanical resources, this study illustrates how digital curation facilitates the transition from traditional empirical knowledge to data-driven therapeutic applications, serving as a valuable resource and foundation for modern drug research.

Despite the development of these resources, certain challenges must be addressed. First, an over-reliance on isolated chemical data often occurs at the expense of ethnobotanical context, which is essential for interpreting the physiological significance of these botanical products. This gap is further exacerbated by linguistic barriers in regional documentation and inconsistent data models that fail to capture the qualitative nuances of traditional medical systems [105, 106]. Second, taxonomic complexities such as synonyms and hybrids frequently compromise the accuracy of downstream research, as many databases fail to account for critical phytochemical variations driven by different origins [107, 108], parts used [109], sizes [110, 111], or cultivation status [112]. This lack of rigorous identification remains a significant barrier to data standardization, rendering the botanical information in many current platforms unreliable for reproducible drug discovery. Third, the insufficient technical exchange between biologists and computational professionals often hinders effective collaboration, particularly when developers lack a foundational understanding of the biological concepts essential for robust database construction [113]. Furthermore, as these databases disseminate knowledge rooted in the traditions of indigenous peoples and local communities, it is crucial to incorporate their intellectual property rights in alignment with the Nagoya Protocol [114, 115]. This stewardship requires upholding the principles of Access and Benefit-Sharing (ABS), ensuring that any benefits, especially from commercial exploitation, are shared equitably with the communities that nurtured this botanical heritage.

Ensuring the long-term validity and authority of these resources requires ongoing maintenance and the timely integration of emerging multi-omics and pharmacological data. Moving forward, the establishment of interoperability standards will be paramount to facilitate comparisons across databases [116], allowing researchers to integrate complementary data sources to address increasingly complex research questions. This ongoing effort to build specialized, interoperable databases, leveraging diverse data types such as pharmacogenomics and systems pharmacology, represents a key strategy for accelerating natural product-based drug development. Through strategic collaboration and data sharing, these optimized resources will ultimately maximize their impact on global health. In conclusion, the systematic evaluation and gap analysis presented in this work establish a foundational bioinformatics framework, effectively unifying ancient botanical wisdom with the rigorous requirements of modern drug discovery.

## Data Availability

No data was used for the research described in the article.

## Abbreviations

ABS: Access and benefit-sharing
ADMET: Absorption, distribution, metabolism, excretion, and toxicity
AI: Artificial intelligence
AYUSH: Ayurveda, Yoga and Naturopathy, Unani, Siddha, Sowa-Rigpa, and Homeopathy
CAM: Complementary and alternative medicine
CARE: Collective benefit, authority to control, responsibility, and ethics
DL: Drug-likeness
EANPDB: Eastern African Natural Products Database
FAIR: Findability, accessibility, interoperability, and reusability
GC-MS: Gas chromatography-mass spectrometry
GIS: Geographic information system
GNDC: Gene-encoded Natural Diverse Components Repository
IR: Infrared spectroscopy
ITM: Iranian traditional medicine
MPNS: Medicinal Plant Names Services
MS: Mass spectrometry
NANPDB: Northern African Natural Products Database
NMR: Nuclear magnetic resonance
OB: Oral bioavailability
QSAR: Quantitative structure–activity relationship
TCM: Traditional Chinese medicine
UV/VIS: Ultraviolet-visible
